# The Plasma Metabolomic Profile is Differently Associated with Liver Fat, Visceral Adipose Tissue, and Pancreatic Fat

**DOI:** 10.1210/clinem/dgaa693

**Published:** 2020-10-30

**Authors:** Lars Lind, Samira Salihovic, Ulf Risérus, Joel Kullberg, Lars Johansson, Håkan Ahlström, Jan W Eriksson, Jan Oscarsson

**Affiliations:** 1 Department of Medical Sciences, Uppsala University, Uppsala, Sweden; 2 School of Medical Sciences, Örebro University, Örebro, Sweden; 3 Department of Public Health and Caring Sciences, Clinical Nutrition and Metabolism, Uppsala University, Uppsala, Sweden; 4 Antaros Medical AB, Gothenburg, Sweden; 5 Department of Surgical Sciences, Radiology, Uppsala University, Uppsala, Sweden; 6 BioPharmaceuticals R&D, AstraZeneca, Gothenburg, Sweden

**Keywords:** ectopic fat, MRI, liver fat, pancreatic fat, metabolomics, obesity

## Abstract

**Context:**

Metabolic differences between ectopic fat depots may provide novel insights to obesity-related diseases.

**Objective:**

To investigate the plasma metabolomic profiles in relation to visceral adipose tissue (VAT) volume and liver and pancreas fat percentages.

**Design:**

Cross-sectional.

**Setting:**

Multicenter at academic research laboratories.

**Patients:**

Magnetic resonance imaging (MRI) was used to assess VAT volume, the percentage of fat in the liver and pancreas (proton density fat fraction [PDFF]) at baseline in 310 individuals with a body mass index ≥ 25 kg/m^2^ and with serum triglycerides ≥ 1.7 mmol/l and/or type 2 diabetes screened for inclusion in the 2 effect of omega-3 carboxylic acid on liver fat content studies.

**Intervention:**

None.

**Main Outcome Measure:**

Metabolomic profiling with mass spectroscopy enabled the determination of 1063 plasma metabolites.

**Results:**

Thirty metabolites were associated with VAT volume, 31 with liver PDFF, and 2 with pancreas PDFF when adjusting for age, sex, total body fat mass, and fasting glucose. Liver PDFF and VAT shared 4 metabolites, while the 2 metabolites related to pancreas PDFF were unique. The top metabolites associated with liver PDFF were palmitoyl-palmitoleoyl-GPC (16:0/16:1), dihydrosphingomyelin (d18:0/22:0), and betaine. The addition of these metabolites to the Liver Fat Score improved C-statistics significantly (from 0.776 to 0.861, *P = *0.0004), regarding discrimination of liver steatosis.

**Conclusion:**

Liver PDFF and VAT adipose tissue shared several metabolic associations, while those were not shared with pancreatic PDFF, indicating partly distinct metabolic profiles associated with different ectopic fat depots. The addition of 3 metabolites to the Liver Fat Score improved the prediction of liver steatosis.

Adipose tissue is a major regulator of energy metabolism and glucose homeostasis that maintains the storage and degradation of triglycerides. Physiologically, storage of adipose tissue will preferentially take place in subcutaneous adipose tissue (SAT). However, in certain individuals and during excess caloric intake, triglycerides and other lipids are also stored in other depots, a condition that denoted ectopic fat storage (1). Ectopic fat storage includes storage in the liver, intra-abdominally (visceral adipose tissue [VAT]), and in the pancreas, as well as other tissues. Ectopic fat in different tissues are suggested to be differentially associated with metabolic risk (2). Importantly, VAT is commonly associated with liver fat, and both depots have not only been suggested to augment the risk of adverse metabolic effects (3, 4), but may also be primary drivers of key metabolic disorders, including dyslipidemia and insulin resistance, either independently of each other (4) or by shared mechanisms. However, little is known how ectopic fat in different tissues translate to changes in whole body metabolism, and how various metabolic pathways are influenced by different ectopic fat depots.

During the recent decade, it has been possible to measure a great number of circulating molecules (<1.5 kDa) involved in intermediary metabolism by mass spectrometry (MS) or nuclear magnetic resonance spectroscopy. Using such metabolomic techniques, a large number of metabolomic changes have been linked to obesity (5, 6). Several studies using a metabolomics approach have reported a link between elevated circulating levels of branch-chained amino acids and liver steatosis (7–11). Other metabolites that have been linked to increased liver fat include dimethylguanidino valeric acid (12), anandamide (13), gamma-glutamyl dipeptides (7), hydroquinone and nicotinic acid (14), high-density lipoprotein measures, and several fatty acids, including long-chain omega-6 fatty acids (9).

In most of the above-cited studies, it is, however, not evaluated if the metabolic changes reported are due to obesity per se or associated ectopic fat distribution.

Few studies have investigated the circulating metabolomic profile associated with increased VAT volume. In 1 study conducted only in subjects with obesity, the group with increased VAT showed significantly higher levels of medium-chain and long-chain acylcarnitines, urobilinogen, docosahexaenoic acid (C22:6ω3), lysophosphoethanolamine (22:6), lysophosphatidylcholine (lysoPC) (22:6), lysoPC (22:5), methoxybenzenepropanoic acid, and isodesmosine (15). In another study using metabolomics to assess metabolomic differences between VAT and SAT, branched-chain amino acids (BCAA) were positively associated with VAT volume, but less so with SAT volume (16). In a recent study of 2 large community-based cohorts, nuclear magnetic resonance spectroscopy metabolomics was used to investigate the associations of metabolites with VAT (17). This study replicated associations of BCAA, but also lactate, glutamine (inversely), and atherogenic lipids were associated with VAT. It has been shown that levels of circulating triglycerides and hemoglobin A1c (HbA1c) could predict pancreatic fat percentage (18). However, to the best of our knowledge, the link between alterations in the metabolome and pancreatic fat percentage has not yet been studied.

In a study of 12 individuals with a mean body mass index (BMI) of 25 kg/m^2^, a high correlation was seen between VAT and intrahepatic fat (correlation coefficient 0.84) (19). A similar high correlation between VAT and intrahepatic fat (0.67) was noted in another study of 23 individuals with a similar mean BMI (20). High correlations between pancreatic fat content and VAT (0.78) and intrahepatic fat content (0.85) have been reported in a sample of individuals with a mean BMI of 26.5 kg/m^2^ (20).

In this study, we hypothesized that 3 ectopic fat depots (VAT volume, liver PDFF and pancreas PDFF) are related to each other, but associated with distinct metabolic profiles. To investigate this, we examined associations of 1063 plasma metabolite levels with measures of VAT volume, and liver fat and pancreatic fat percentages. Another aspect we aimed to investigate was the detection of nonalcoholic fatty liver disease (NAFLD), which is with current methods expensive and cumbersome, either demanding histology or imaging. A simple test based on biochemistry and other physical variables might be a tool to select patients to be referred to MRI or histology. Although several noninvasive prediction scores for NAFLD have been developed (21–24), the C-statistics for those scores are not optimal, being in the range of 0.80–0.83. Therefore, we evaluated whether the addition of the metabolites associated with liver fat in the present study would improve the prediction of a validated score for NAFLD, the NAFLD Liver Fat Score (LFS) (23).

## Material and Methods

### Study population and sample collection

Data from 140 patients from the EFFECT I study and 170 from the EFFECT II study in whom a successful abdominal MRI scan was performed were used together in a unified sample. In the EFFECT I study (NCT02354976), patients were eligible for screening for inclusion in the treatment part of the study if they were 40–75 years old and had a BMI of 25 to 40 kg/m^2^, serum triglycerides levels of ≥ 1.7 mM (150 mg/dL), and liver PDFF > 5.5%. Exclusion criteria included having diabetes mellitus, a history of other hepatic disease, an inability to undergo MRI scanning, and a significant alcohol intake (over 14 units per week) (25). In the EFFECT II study (NCT02279407) patients were eligible for screening for inclusion in the treatment part of the study if they were 40–75 years old and had a BMI of 25 to 40 kg/m^2^, a history of type 2 diabetes mellitus, and liver PDFF > 5.5%. Exclusion criteria included a history of other hepatic disease, inability to undergo MRI scanning, and a significant alcohol intake (over 14 units per week) (26). Only baseline screening data from both randomized and nonrandomized patients from the EFFECT I and II studies were used in the present study. The participants were investigated in the morning after an overnight fast. Blood samples were taken in the morning and analyses were performed as previously described (23, 24). A weight-scale with bioimpedance (Tanita, Tokyo, Japan) was used for determinations of body weight and total body fat. Further details on the methods in the EFFECT I and II studies have previously been published (25, 26).

### Metabolomics

The nontargeted metabolomic analysis was performed at Metabolon, Inc. (Morrisville, North Carolina). All plasma samples were stored at -80°C until processed. The samples were extracted with methanol and the supernatants divided into 5 equal fractions: 2 for analysis by 2 separate reverse phase (RP)/ultraperformance liquid chromatography–tandem mass spectrometry (UPLC-MS/MS) methods with positive ion mode electrospray ionization (ESI) optimized for more hydrophilic and hydrophobic compounds respectively, one for analysis by RP/UPLC-MS/MS with negative ion mode ESI, 1 for analysis by hydrophilic interaction liquid chromatography/UPLC-MS/MS with negative ion mode ESI, and 1 sample was reserved for backup (27). All methods utilized a Waters ACQUITY UPLC and a Thermo Scientific Q-Exactive high resolution/accurate mass spectrometer interfaced with a heated electrospray ionization (HESI-II) source and Orbitrap mass analyzer operated at 35 000 mass resolution. The MS analysis alternated between MS and data-dependent MS^n^ scans using dynamic exclusion. The scan range varied slightly between methods but covered 70 to1000 m/z. Compounds were identified by comparison to library entries of purified standards or recurrent unknown entities based on retention time, molecular weight, preferred adducts, and in-source fragments, as well as associated MS spectra and curated by visual inspection for quality control using proprietary software developed by Metabolon (https://www.intechopen.com/books/metabolomics/software-techniques-for-enabling-high-throughput-analysis-on-metabolomic-datasets). The method for which each metabolite is quantitated is dependent on factors such as interference by neighboring peaks and reproducibility/variability. Metabolites that were not annotated by using the in-house library of standards were given a number.

### Imaging

Magnetic resonance imaging was used to determine liver PDFF using the median of the fat fraction values inside the delineated total liver volume, and the visceral adipose tissue volume in the abdominal depot with scan centered at the L4/L5 vertebrae, as previously described (25, 26). The pancreas was segmented by a trained operator from the axial slices of the water image using ImageJ software. The border of the pancreas was avoided to reduce partial volume effects. Proton density fat fraction of the pancreas was determined using the median of the fat fraction values inside the delineated pancreas volume.

### Liver fat score

We used the LFS, since this score for detection of NAFLD used magnetic resonance spectroscopy, and not ultrasound, for the definition of NAFLD (23). The NAFLD LFS formula (23): -2.89 + (1.18*MetS, 0 for absence and 1 for MetS) + (0.45*type 2 diabetes, 0 for absence and 2 for type 2 diabetes) + (0.15*insulin) + (0.04* aspartate aminotransferase) - (0.94*aspartate aminotransferase/ alanine aminotransferase), where MetS is the metabolic syndrome according to the international diabetes federation criteria (insulin in mU/l and liver enzymes in µkat/l) (28).

### Statistical methods

The measurements of the 3 ectopic fat depots, VAT, liver and pancreatic PDFF, were ln-transformed to achieve normal distributions in the analysis.

**Figure 1.**
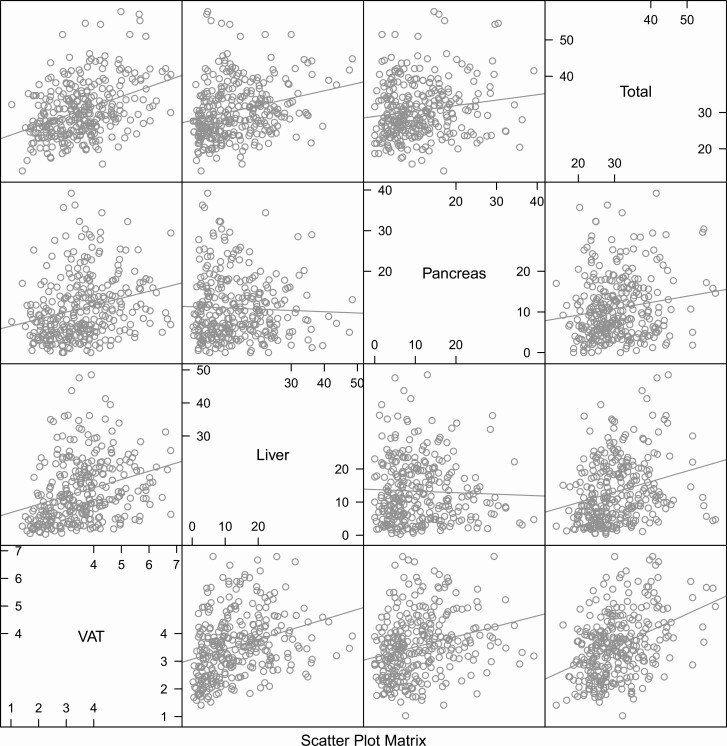
Scatterplot matrix for total fat mass and the three ectopic fat depots; visceral adipose tissue (VAT), liver and pancreatic proton density fat fraction (PDFF). Total fat, liver and pancreatic PDFF are given in %, while VAT is given as L.

First, the relationships between the 3 ectopic fat depots and total fat mass were visualized (Fig. 1) by pairwise correlation analysis using Pearson´s correlation coefficient.

Second, the relationships between the 3 fat depots and traditional lipid measurements were performed by linear regression models (serum triglycerides ln-transformed). Adjustment was performed for age, sex, fat mass, and plasma glucose as confounders.

Third, we evaluated each of the 1063 metabolites 1 by 1 (as dependent variables) in relation to the 3 ectopic fat depots (1 by 1 as independent variables), with age, sex, glucose (ln-transformed values), and total fat mass as confounders. The original metabolomics data were used and subjected to inverse rank normalization in order to achieve a normal distribution for the metabolites. Missing values for the metabolites were not imputed.

The analyses were performed in a discovery step (a random 2/3 of the sample) and a validation step (the remaining 1/3 of the sample), where only the metabolites showing a false discovery rate < 0.05 in the discovery step were validated. In the validation step, *P < *0.05 was considered to be significant.

We also performed a further adjustment for the levels of circulating lipoproteins. Furthermore, in separate models, we included interaction terms between diabetes and the fat depots in order to evaluate if the metabolic profiles associated with different fat depots were different between individuals with and without diabetes.

Fourth, for the validated metabolites described in Table 1, we performed a stepwise linear regression analysis for each of the metabolites as dependent variables and with VAT, liver PDFF, and pancreatic PDFF as independent variables together with the confounders of age, sex fasting glucose, and fat mass (confounders being forced into the model). Entry into, as well as deletion from, the models were both set to *P < *0.05. In these exploratory analyses, the total dataset was used and *P < *0.05 was regarded as significant.

**Table 1.** Relationships between metabolites and liver PDFF, VAT volume and Pancreas PDFF identified by a split-sample, discovery/validation approach

Metabolite	Beta (95% CI)	*P*-value Adjusted for Age, Sex Fat Mass, and Fasting Glucose	*P*-value Adjusted for Triglycerides, LDL-, and HDL- Cholesterol
**Liver PDFF**			
palmitoyl-oleoyl-glycerol (16:0/18:1) [1]^*a*^	0.66 (0.42, 0.89)	2.09e-06	0.0276535
palmitoyl-oleoyl-glycerol (16:0/18:1) [2]^*a*^	0.51 (0.26, 0.75)	0.0001974	0.099461
behenoyl dihydrosphingomyelin (d18:0/22:0)^*a*^	0.35 (0.16, 0.55)	0.0006821	0.0001556
alanine	0.31 (0.13, 0.48)	0.0009926	0.0096519
guanidinosuccinate	-0.32 (-0.51, -0.14)	0.0010368	0.0013787
1-stearoyl-2-oleoyl-GPC (18:0/18:1)	0.3 (0.11, 0.49)	0.0021801	0.0195459
palmitoyl-linoleoyl-glycerol (16:0/18:2) [2]^*a*^	0.39 (0.1, 0.67)	0.0104842	0.900037
X—24585	-0.28 (-0.48, -0.07)	0.0110406	0.108352
X—17359	0.26 (0.06, 0.46)	0.0127735	0.0068602
cysteine-glutathione disulfide	-0.24 (-0.43, -0.05)	0.0133266	0.0672796
diacylglycerol (14:0/18:1, 16:0/16:1) [2]^*a*^	0.35 (0.09, 0.62)	0.0135518	0.3935354
1-palmitoyl-2-oleoyl-GPE (16:0/18:1)	0.22 (0.05, 0.39)	0.014054	0.5834851
gamma-glutamylphenylalanine	0.24 (0.05, 0.43)	0.0164097	0.0743783
X—13844	-0.23 (-0.42, -0.04)	0.0194767	0.0030149
isoleucine	0.25 (0.04, 0.46)	0.0197214	0.0289615
pimeloylcarnitine/3-methyladipoylcarnitine (C7-DC)	-0.36 (-0.66, -0.07)	0.0212874	0.0424094
1-palmitoyl-2-palmitoleoyl-GPC (16:0/16:1)^*a*^	0.23 (0.04, 0.43)	0.0227848	0.0931553
3-methyl-2-oxovalerate	0.27 (0.04, 0.51)	0.0236417	0.0294755
1-palmitoleoylglycerol (16:1)^*a*^	0.27 (0.04, 0.5)	0.0253739	0.1310313
1-stearoyl-2-oleoyl-GPE (18:0/18:1)	0.21 (0.03, 0.39)	0.0255566	0.9880193
palmitoleoyl-oleoyl-glycerol (16:1/18:1) [2]^*a*^	0.28 (0.04, 0.52)	0.0258783	0.6679497
1-stearoyl-2-linoleoyl-GPE (18:0/18:2)^*a*^	0.21 (0.02, 0.39)	0.0291936	0.9942857
1-(1-enyl-palmitoyl)-2-oleoyl-GPC (P-16:0/18:1)^*a*^	-0.19 (-0.37, -0.02)	0.0328427	0.282608
3-(4-hydroxyphenyl)lactate	0.29 (0.02, 0.55)	0.0370505	0.1035482
palmitoyl-linoleoyl-glycerol (16:0/18:2) [1]^*a*^	0.3 (0.02, 0.58)	0.0404118	0.5825614
X—12063	0.2 (0.01, 0.39)	0.0409261	0.142359
X—17340	0.22 (0.01, 0.43)	0.0410367	0.0439835
glycine	-0.2 (-0.38, -0.01)	0.0414147	0.0590888
pantothenate (Vitamin B5)	-0.31 (-0.6, -0.02)	0.0420067	0.0335255
salicylate	-0.21 (-0.41, -0.01)	0.0473171	0.0186942
3-methyl-2-oxobutyrate	0.24 (0, 0.47)	0.049219	0.0096713
**Pancreas PDFF**			
5-hydroxylysine	0.24 (0.03, 0.45)	0.0249613	0.0182769
taurodeoxycholate	0.21 (0, 0.41)	0.0481979	0.0571838
**VAT**			
X—21659	0.29 (0.14, 0.44)	0.0003152	0.0012897
5-methylthioadenosine (MTA)	0.25 (0.1, 0.39)	0.0013369	0.0407127
N6-succinyladenosine	0.29 (0.12, 0.47)	0.0013831	0.0135583
X—15492	0.27 (0.11, 0.44)	0.0019693	0.0021252
erythronate^*a*^	0.31 (0.11, 0.5)	0.0027191	0.0097656
X—13529	0.26 (0.09, 0.43)	0.0036443	0.0806253
N-acetylisoleucine	0.28 (0.09, 0.46)	0.0042548	0.0166546
O-sulfo-L-tyrosine	0.22 (0.07, 0.37)	0.0057188	0.0092753
gamma-glutamylisoleucine^*a*^	0.25 (0.07, 0.43)	0.006827	0.022808
X—12718	0.24 (0.07, 0.41)	0.0072968	0.0343109
1-palmitoyl-2-oleoyl-GPC (16:0/18:1)	0.24 (0.05, 0.42)	0.0130278	0.0024127
alpha-ketoglutarate	0.25 (0.05, 0.44)	0.0141122	0.0235949
X—18889	0.25 (0.05, 0.44)	0.0142308	0.1130927
X—15497	0.23 (0.05, 0.41)	0.0142313	0.1736486
X—22102	0.21 (0.04, 0.38)	0.0154009	0.2451702
1-(1-enyl-palmitoyl)-2-oleoyl-GPC (P-16:0/18:1)^*a*^	-0.18 (-0.32, -0.04)	0.0159883	0.4521156
2-methylbutyrylcarnitine (C5)	0.26 (0.05, 0.47)	0.0195666	0.3626207
X—17359	0.2 (0.03, 0.36)	0.0197337	0.0045845
o-cresol sulfate	0.21 (0.04, 0.39)	0.0214102	0.2540891
1-palmitoyl-2-palmitoleoyl-GPC (16:0/16:1)^*a*^	0.19 (0.02, 0.35)	0.0260116	0.0352729
X—11564	0.18 (0.02, 0.33)	0.0263369	0.1254097
1-(1-enyl-palmitoyl)-2-linoleoyl-GPC (P-16:0/18:2)^*a*^	-0.15 (-0.29, -0.02)	0.031965	0.7779452
gamma-glutamylvaline	0.18 (0.02, 0.34)	0.0328581	0.2096443
3-hydroxy-3-methylglutarate	0.2 (0.02, 0.39)	0.0367995	0.0453413
X—12063	0.17 (0.01, 0.32)	0.0394836	0.133079
5alpha-androstan-3beta,17beta-diol disulfate	0.17 (0.01, 0.33)	0.0440436	0.0733401
2-aminoadipate	0.18 (0.01, 0.35)	0.0451287	0.0511283
X—23587	0.17 (0.01, 0.33)	0.0457273	0.0553625
X—12846	0.15 (0, 0.3)	0.049467	0.0239679
imidazole propionate	0.15 (0, 0.3)	0.0498133	0.1781602

The relationships were adjusted for age, sex, fasting glucose, and total fat mass, as well as further adjusted also for lipoprotein levels (serum triglycerides and HDL- and LDL-Cholesterol). Only metabolites with a *P*-value ≤ 0.05 in the validation step are shown. Abbreviations: CI, confidence interval; HDL, high-density lipoprotein; LDL, low-density lipoprotein; PDFF, proton density fat fraction; VAT, visceral adipose tissue.

^*a*^indicates that there is no available standard; [1] or [2] indicate a compound with 2 distinct peaks (isomers) and which of the 2 that was detected.

Fifth, improvement in discrimination of NAFLD (PDFF > 5.5%) used the comparison between 2 logistic regression models with NAFLD as the binary outcome. We first screened the top 10 metabolites regarding liver PDFF in logistic regression models to assess which metabolites are being associated with NAFLD when added to LFS. We also took their inter-relationships into account. To evaluate any improvement in discrimination, we then compared a model only using LFS as the independent variable and a second model also including the metabolites found to be significantly related to liver PDFF in the screening. C-statistics were used to calculate if the addition of the metabolites to LFS improved discrimination.

STATA14 (Stata Inc., College Station, Texas) was used for the calculations, while the graphics were also created by R 3.4 (lattice package).

### Metabolic pathway analysis

Further interpretation of the set of metabolites significantly associated with the 3 ectopic fat depots was performed using MetaboAnalyst 4.0. The analysis combines pathway enrichment analysis with pathway topology in order to simplify biological interpretation and pathway visualization (29). Metabolites from curated human metabolic pathways (Kyoto Encyclopedia of Genes and Genomes) were included in an overrepresentation analysis, which uses a hypergeometric test to evaluate whether the metabolite set of interest is represented more than expected by chance within that specific list. In this case the 3 different sets including only the annotated metabolites (VAT: 18/30 annotated; liver PDFF: 26/31 annotated; pancreatic PDFF: 2/2 annotated) were tested. Thereafter a pathway topology analysis using relative-betweeness centrality was performed to discern metabolic network patterns.

## Results

The clinical characteristics, including VAT volume, liver PDFF, and pancreas PDFF are shown in Table 2. The participants were 64.6 (SD 7.2) years: 39% were female and 43% of the sample had type 2 diabetes. The mean BMI was 30.4 kg/m^2^ (SD 3.4). The mean VAT volume was 3.5 L (SD 1.1) and the mean percentage of total fat, liver PDFF, and pancreas PDFF was 33.8 % (SD 7.8), 13.2 % (SD 9.5), and 10.8 % (SD 7.7), respectively. The prevalence of NAFLD defined as liver PDFF > 5.5% was 74%.

**Table 2.** Basic characteristics of the study population (n = 310)

Characteristic	Mean (SD) or proportion (%)
Age	64.6 (7.2)
Sex (% female)	39
Systolic blood pressure (mmHg)	143 (17)
Weight (kg)	90 (13)
Height (cm)	172 (9)
Waist circumference (cm)	107 (11)
BMI (kg/m^2^)	30.4 (3.4)
Diabetes medication (%)	41
Statin treatment (%)	39
Antihypertensive treatment (%)	57
Fasting glucose (mmol/l)	7.4 (2.0)
Fasting insulin (mU/l)	10.9 (6.9)
Serum cholesterol (mmol/l)	5.51 (1.41)
Serum triglycerides (mmol/l)	2.14 (1.16)
HDL-cholesterol (mmol/l)	1.34 (0.37)
LDL-cholesterol (mmol/l)	3.47 (1.23)
Liver PDFF (%)	13.2 (9.5)
Pancreas PDFF (%)	10.8 (7.7)
VAT (L)	3.5 (1.1)
Total body fat (%)	33.8 (7.3)

Abbreviations: BMI, body mass index; HDL, high-density lipoprotein; LDL, low-density lipoprotein; PDFF, proton density fat fraction; SD, standard deviation; VAT, visceral adipose tissue.

### Correlations between fat depots

As shown in Fig. 1, total body fat mass and the amounts of 3 different ectopic fat depots were all significantly related to each other pairwise, except for liver versus pancreatic fat percentages being nonsignificant. In Fig. 1, the raw data of all variables are given. When the VAT, liver PDFF, and pancreatic PDFF were ln-transformed, the correlation coefficients were in the 0.25 to 0.40 range (*P < *0.001) for all pairwise relationships.

### Serum lipoproteins vs fat depots

Table 3 shows associations of the 3 ectopic fat depots with clinically measured serum lipids: total cholesterol, triglycerides, high-density lipoprotein (HDL)-cholesterol, and low-density lipoprotein (LDL)-cholesterol. Following adjustment for age, sex, total body fat mass, and glucose, VAT volume was positively associated with serum triglycerides but negatively associated with total cholesterol, HDL,- and LDL-cholesterol. Liver PDFF was positively associated with serum triglycerides and negatively associated with HDL-cholesterol, while pancreas PDFF was negatively associated with LDL-cholesterol.

**Table 3.** Relationships between traditional lipids and three depots of ectopic fat accumulation following adjustment for age, sex, fat mass, and glucose

Lipid (mmol/L)	VAT (L)		Liver PDFF (%)		Pancreas PDFF (%)	
	**Beta (95% CI)**	***P*-value**	**Beta (95% CI)**	***P*-value**	**Beta (95% CI)**	***P*-value**
Total cholesterol	-121 (-210, -32)	0.0083	-0.6 (-1.4, 0.19)	0.14	-0.62 (-1.24, 0.01)	0.054
Serum triglycerides	121 (15, 228)	0.026	1.73 (0.8, 2.66)	0.0003	-0.02 (-0.77, 0.73)	0.95
HDL-cholesterol	-746 (-1075, -417)	0.0001	-5.87 (-8.83, -2.92)	0.0001	-0.13 (-2.53, 2.28)	0.92
LDL-cholesterol	-125 (-227, -24)	0.016	-0.69 (-1.59, 0.21)	0.13	-0.76 (-1.47, -0.06)	**0.035**

Abbreviations: CI, confidence interval; HDL, high-density lipoprotein; LDL, low-density lipoprotein PDFF, proton density fat fraction; VAT, visceral adipose tissue.

### Metabolomic profiles associated with VAT, liver PDFF, and pancreatic PDFF

The discovery/replication analysis showed that 59 metabolites were found to correlate with ectopic fat distribution of the 3 depots: VAT, liver, and pancreas. Table 1 shows the metabolites (including unknown metabolites) adjusted for age, sex, fasting glucose, and total fat mass.

Following adjustment for age, sex, total fat mass, and glucose, 30 metabolites were correlated with VAT volume, out of which 18 were annotated. Thirty-one metabolites were correlated with liver PDFF, out of which 26 were annotated. Two annotated metabolites were correlated with pancreas PDFF. The 2 metabolites identified to be associated with pancreas PDFF were not associated with the other 2 ectopic fat depots, while liver PDFF and VAT volume shared 4 significant metabolites (2 unknown metabolites, 1-(1-enyl-palmitoyl)-2-oleoyl-GPC (P-16:0/18:1) and 1-palmitoyl-2-palmitoleoyl-GPC (16:0/16:1)).

Following further adjustment for serum lipoprotein levels, 15 metabolites were correlated with VAT, while 14 were associated with liver PDFF. Only 1 metabolite (5-hydroxylysine) was associated with pancreas PDFF following adjustment for serum lipoprotein levels.

Including interaction terms between diabetes and the fat depots (data not shown) did not support that the metabolic profiles associated with different fat depots were different between individuals with and without diabetes.

Using a stepwise procedure to investigate if any of the other ectopic fat depots contributed to the results presented in Table 1, none of the other 2 fat depots showed *P < *0.05 for the relationships found versus liver PDFF.

For relationships found versus pancreatic PDFF, 5-hydroxylysine was also related to liver fat (*P = *0.04).

For relationships found versus VAT, liver PDFF was related to 5-methylthioadenosine (MTA) (*P = *0.05 for liver PDFF). Also, pancreatic PDFF was related to imidazole propionate (*P = *0.01), and regarding the unidentified metabolite X–13 529, relationships were seen for VAT volume, as well as for liver PDFF (*P < *0.01) and pancreatic PDFF (*P < *0.04).

### Metabolic pathways associated with VAT, liver PDFF, and pancreatic PDFF

**Figure 2.**
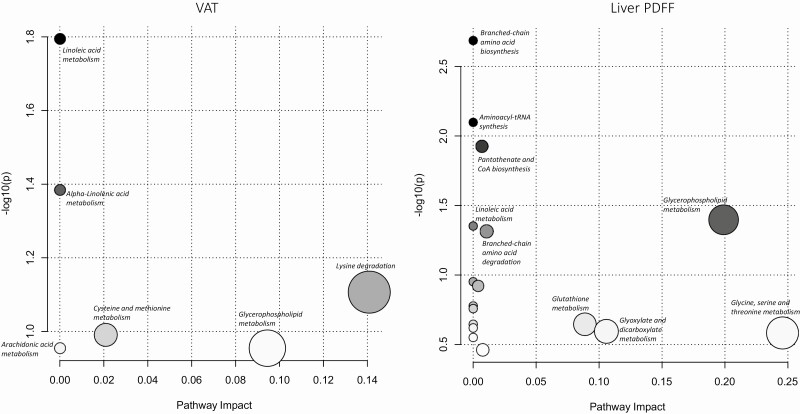
Metabolic pathways significantly associated with both visceral adipose tissue (VAT) volume and liver proton density fat fraction (PDFF). The metabolites are arranged according to the scores based on enrichment analysis (y-axis) and topology analysis (x-axis). The color and size of each circle are based on *P*-values and pathway impact values, respectively.

Pathway analyses for each fat depot were performed using overrepresentation, pathway enrichment, and pathway topology analysis. Five metabolic pathways were identified to map to the metabolite set significantly associated with VAT (Fig. 2). Two of these pathways showed significant associations with VAT (using *P*-values obtained from pathway enrichment analyses reflecting the overall association of the VAT metabolite set) and included linoleic acid metabolism (*P = *0.016, -log[*p*]* = *4.13) and alpha-Linolenic acid metabolism (*P = *0.0413, -log[*p*]* = *3.19). Seventeen metabolic pathways were identified to map the metabolite set significantly associated with liver PDFF (Fig. 2). Six of these pathways showed significant associations with liver PDFF, and the strongest associations were observed for BCAA biosynthesis (*P = *0.00206, -log[*p*]* = *6.19) and aminoacyl-tRNA biosynthesis (*P = *0.00798, -log[*p*]* = *4.83). Only the metabolic pathway of lysine degradation showed significant associations with pancreatic PDFF (*P = *0.0320, -log[*p*]* = *3.44).

### Liver fat score discrimination improvement

**Figure 3.**
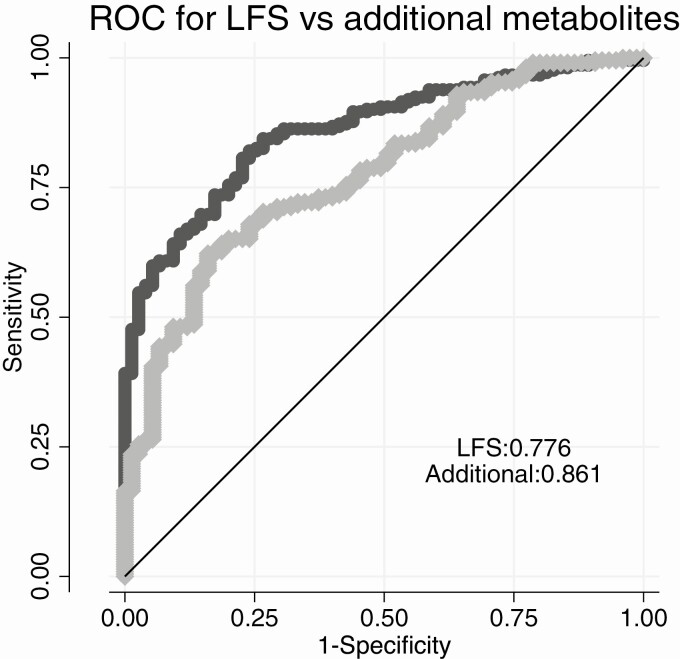
Area under the curve (AUC) for the liver fatty score (LFS) alone (light grey) and when 3 metabolites were added (1-palmitoyl-2-palmitoleoyl-GPC (16:0/16:1), behenoyl dihydrosphingomyelin (d18:0/22:0) and betaine) (dark grey) regarding discrimination of nonalcoholic fatty liver disease (NAFLD) in the sample.

The mean LFS was 4.27 (SD 2.44). Using a cutoff of -0.64 to define NALFD resulted in 98% of the sample as being predicted to have NAFLD (while 74% had verified NAFLD). Table 4 shows the top 10 metabolites associated with liver fat percentage. Following an analysis of the inter-relationships between these 10 metabolites and how they related to NAFLD (liver PDFF > 5.5%), we evaluated if the top 3 metabolites (1-palmitoyl-2-palmitoleoyl-GPC [16:0/16:1], behenoyl dihydrosphingomyelin [d18:0/22:0], and betaine) added predictive power when added to LFS. As shown in Fig. 3, addition of these 3 metabolites to LFS improved C-statistics significantly (from 0.776 to 0.861, *P = *0.0004).

**Table 4.** The top ten metabolites associated with the liver PDFF following adjustment for age, gender, fat mass, and fasting glucose, as well as further adjusted for lipoprotein levels (serum triglycerides, HDL-, and LDL-cholesterol)

Metabolite	Beta (95% CI)	*P*-value Adjusted for Age, Sex, Fat Mass, and Fasting Glucose	*P*-value Adjusted for Triglycerides, LDL-, and HDL- Cholesterol
1-palmitoyl-2-palmitoleoyl-GPC (16:0/16:1)^*a*^	0.47 (0.34, 0.6)	4.02e-11	4.17e-10
behenoyl dihydrosphingomyelin (d18:0/22:0)^*a*^	0.35 (0.22, 0.47)	1.56e-07	2.79e-08
betaine	-0.35 (-0.48, -0.22)	5.56e-07	5.16e-07
1-palmitoyl-2-oleoyl-GPI (16:0/18:1)^*a*^	0.36 (0.22, 0.5)	7.31e-07	3.24e-06
X—19438	0.37 (0.22, 0.52)	3.67e-06	0.0000244
lactate	0.36 (0.21, 0.5)	3.80e-06	0.0001229
X—24295	0.33 (0.19, 0.47)	4.71e-06	0.0001338
1-palmitoyl-2-oleoyl-GPE (16:0/18:1)	0.32 (0.19, 0.45)	4.74e-06	0.0105331
1-stearoyl-2-oleoyl-GPE (18:0/18:1)	0.31 (0.18, 0.45)	0.0000125	0.0204211
1-palmitoyl-2-arachidonoyl-GPI (16:0/20:4)^*a*^	0.36 (0.2, 0.51)	0.0000126	0.00052

Abbreviations: CI, confidence interval; HDL, high-density lipoprotein; LDL, low-density lipoprotein; PDFF, proton density fat fraction.

^*a*^ Indicates that there is no available standard.

## Discussion

### Principal findings

The main aim of this study was to identify circulating metabolites (and related metabolic pathways) in relation to ectopic fat deposition by use of a large number of plasma metabolites. First, we assessed if specific metabolites were related to VAT volume and liver and pancreatic fat percentages. For this purpose, we adjusted for total body fat mass, since obesity is related to major deviations in the metabolome (5, 6), and we wanted to identify metabolic profiles that were distinctly related to ectopic fat accumulation. We also adjusted for fasting glucose, since we do have a large proportion of patients with type 2 diabetes in the sample and did not want to only identify a metabolic signature being linked to diabetes. We found that the metabolomic profiles of liver fat percentage and VAT were related to a certain extent, but the metabolites associated with pancreatic fat percentage were unique.

We also assessed whether the metabolites associated with liver fat could improve the discriminatory power of the LFS for detection of liver steatosis. We could show that the addition of 3 metabolites to an established score to detect NAFLD (LFS) did markedly increase the discriminatory power by 8.5%.

### Visceral adipose tissue and liver fat associated common metabolites

Visceral adipose tissue and liver PDFF were associated with a similar number of metabolites, but liver PDFF-associated metabolites mapped stronger to metabolic pathways than VAT. Of the annotated metabolites, 2 glycerophosphocholines were significantly related to both VAT and liver PDFF. This finding was supported by pathway analysis, showing that glycerophospholipid metabolism was linked to both VAT and liver PDFF. The pathway analysis also showed associations between linoleic acid metabolism and both VAT and liver PDFF, although the impact was not as great as for glycerophospholipid metabolism. Four metabolites overlapped among VAT and liver fat, and the remaining metabolites were unique, indicating distinct metabolic profiles between VAT volume and liver PDFF.

In previous metabolomics studies (7, 8, 11), positive associations between the BCAAs leucine and isoleucine have been linked to both VAT and liver fat. In the present study, isoleucine was only significantly related to liver PDFF. Since isoleucine was associated also with VAT if not adjusted for total fat mass, this adjustment is possibly the reason for the discrepancy. Furthermore, leucine degradation was 1 of the major pathways associated with VAT, and N-acetylisoleucine were 1of the metabolites linked to VAT, suggesting that BCAAs were also related to VAT in this cohort. Mechanistically, the role of BCAA in metabolic dysfunction has been suggested to be driven, at least in part, by interactions of BCAA with cellular lipid uptake that promotes insulin resistance (30). Also, genes linked to an impaired metabolism of BCAA (31), as well as interactions with gut microbiota characterized by an increased potential for BCAA biosynthesis, have been shown to be associated with insulin resistance (32). Thus, it is likely that the associations with BCAA observed in the present study are more indicative of a general metabolic dysfunction, such as insulin resistance, and not markers of a specific ectopic fat deposition.

A recent study from the UK Biobank cohort suggested a complexity of associations between fat distribution and metabolic status (33). In that study, different diseases (eg, type 2 diabetes and coronary heart disease) were linked to different ectopic fat depots in a complex fashion. In the present study, we find that the plasma metabolome is linked to different ectopic fat depots in a complex fashion, just as with respect to overt diseases.

### Visceral adipose tissue–associated metabolites

The identified top metabolite being related to VAT was 5’-methylthioadenosine, also known as MTA or thiomethyladenosine. 5’-methylthioadenosine is involved in enzymatic reactions and has been linked to the regulation of gene expression, proliferation, differentiation, and apoptosis (34). Also, another adenosine-containing metabolite (N6-succinyladenosine) was amongst the top findings for VAT.

One large study recently found BCAA, lactate, glutamine, and atherogenic lipids to be associated with VAT after adjustment for glucose level, waist circumference, and serum triglycerides (17). We did not identify lactate amongst the metabolites being associated with VAT, but 2 glutamates, which could be converted to glutamine was amongst the metabolites being associated with VAT.

Metabolomics has also implicated elevated VAT with alterations in levels of both long-chain and medium-chain acylcarnitines (15). Elevated levels of 2-methylbutyrylcarnitine have been observed in rare metabolic disorders linked to isobutyryl-coenzyme A dehydrogenase deficiency, in which the body is unable to catabolize BCAA (leucine, isoleucine, valine). However, since only 2-methylbutyrylcarnitine out of the 59 carnitine-related measured metabolites was significantly associated with VAT, our results suggest weak relations between plasma acylcarnitines and the 3 ectopic fat depots evaluated.

### Pancreatic fat associated metabolites

In the present study, pancreatic fat was positively associated with only 2 metabolites, one lysine-derivate and the bile acid conjugate taurodeoxycholate. To our knowledge, no previous studies have assessed global metabolomic profiles regarding pancreatic fat.

### Liver fat score assessment

We found that adding 1-palmitoyl-2-palmitoleoyl-GPC (16:0/16:1), behenoyl dihydrosphingomyelin (d18:0/22:0), and betaine to LFS improved discrimination of NAFLD. Dihydrosphingomyelin (d18:0/22:0) belongs to the class of sphingolipids that are structural components of cell membranes and likely associated with lipoproteins in the circulation. Sphingolipids are bioactive signaling molecules that modulate growth regulation, cell migration, apoptosis, senescence, and inflammatory responses. Experimental studies have implicated plasma sphingolipids in development of liver steatosis (35). Although dihydrosphingomyelin (d18:1/22:0) was closely related to liver fat percentage and could improve the discrimination of NAFLD, the derivation of a score in 1 study population sample always induces a certain amount of overfitting and demands validation in other populations.

The LFS was constructed in a sample with less individuals with type 2 diabetes or the metabolic syndrome as compared with the present sample. This might explain why the LFS overestimated the prevalence of NAFLD in the sample, since LFS was constructed for less metabolically affected patients. Therefore, it is of importance that our finding that the addition of the 3 metabolites to LFS improved discrimination should also be validated in other, less metabolically affected patients.

### Strengths and limitations

The strength of the present study is that we have measured a large number of metabolites in a sample with state-of-the-art MRI measurements of 3 different fat depots. A limitation is that we do not have another independent sample for replication, and therefore used the split sample technique within the same sample for validation. Another limitation is that we used only an overweight/obese group with a high risk of NAFLD and increased VAT volume and pancreatic fat percentage, so the results have to be reproduced in population-based samples in order to be generalizable. On the other hand, the current population with a higher amount of ectopic fat overall might be a highly relevant target group to study these relationships.

We could not find correlations that were as close between the measurements of the ectopic fat depots, as previously presented (19). This was especially seen for pancreatic PDFF not being significantly related to liver PDFF. The pancreas was manually segmented, while a computerized algorithm was used for the liver. It is well-known that it is harder to distinguish the borders of the pancreas as compared with the liver, and therefore the values of pancreatic PDFF are less reproducible (36). This might also explain why we find less significant correlations between pancreatic PDFF and the metabolites as well as versus lipoprotein levels. This should be acknowledged as a limitation of the study.

Menopause could alter the metabolic status and fat deposition in women. We did not take menopause in consideration in this study since we do not have this information in our database. However, since only a handful of the women included were in the age of still having menstruations, we do not expect any major influence of menopause on the results presented.

In the “-omics” era of exploring multiple biomarkers at the same time, there is always a trade-off between not reporting false positive findings and not producing too many false negative results. We have previously used the discovery/validation approach with a false discovery rate < 0.05 in the discovery step and *P < *0.05 in the validation step, since statistical simulations on this matter have justified that approach (37).

In conclusion, we show that liver fat percentage and VAT have several overlapping metabolomic associations, while those were not shared with pancreatic fat percentage. However, we also identified unique metabolomic associations for liver fat percentage and VAT, respectively, suggesting partly different metabolic changes associated with these 2 ectopic fat depots. We could also show that the addition of 3 metabolites to the established LFS score to detect NAFLD significantly improved the discrimination regarding this condition.

## Data Availability

Data underlying the findings described in this manuscript may be available upon request in accordance with AstraZeneca’s data sharing policy described at https://astrazenecagroup-dt.pharmacm.com/DT/Home. Please send requests to: jan.oscarsson@astrazeneca.com.

## Additional Information

***Disclosure Summary:*** Jan Oscarsson is employed by AstraZeneca. Lars Lind and Ulf Riserus served as consultants for AstraZeneca regarding the EFFECT I and II studies. Jan Eriksson has received research grants or honoraria from AstraZeneca, NovoNordisk, Merck Sharpe, and Dohme and Mundipharma. Joel Kullberg, Lars Johansson, and Håkan Ahlström are co-founders, co-owners, and employed at Antaros Medical AB.
